# Glymphopathy and Reduced Processing Speed in Community-Dwelling Adults with Silent Cerebral Small Vessel Disease: A DTI-ALPS Study

**DOI:** 10.3390/jcm14176039

**Published:** 2025-08-26

**Authors:** Zaw Myo Hein, Muhammad Faqhrul Fahmy Arbain, Muhammad Danial Che Ramli, Usman Jaffer, Che Mohd Nasril Che Mohd Nassir

**Affiliations:** 1 Department of Basic Medical Sciences, College of Medicine, Ajman University, Ajman P.O. Box 346, United Arab Emirates; z.hein@ajman.ac.ae; 2 Department of Anatomy and Physiology, School of Basic Medical Sciences, Faculty of Medicine, Universiti Sultan Zainal Abidin, Kuala Terengganu 20400, Malaysia; faqhrulfahmy@gmail.com; 3 Department of Diagnostic and Allied Health Science, Faculty of Health and Life Sciences, Management and Science University, Shah Alam 40100, Malaysia; muhddanial_cheramli@msu.edu.my; 4 Kulliyyah of Islamic Revealed Knowledge and Human Sciences, International Islamic University Malaysia, Kuala Lumpur 50728, Malaysia; jafferu@iium.edu.my

**Keywords:** cerebral small vessel disease, enlarged perivascular spaces, glymphatic system, processing speed, diffusion tensor imaging

## Abstract

**Background/Objectives**: Cerebral small vessel disease (CSVD) often manifests as enlarged perivascular spaces (ePVS), which are linked to reduced processing speed even in asymptomatic individuals. Glymphatic dysfunction (or glymphopathy) has been proposed as a mechanism underlying ePVS, with the diffusion tensor image analysis along the perivascular space (DTI-ALPS) index serving as a potential non-invasive surrogate marker. This study aimed to examine the association between DTI-ALPS index, ePVS burden, and processing speed in community-dwelling adults without overt neurological symptoms, stratified by QRISK3 cardio-cerebrovascular risk prediction score. **Methods**: Sixty young-to-middle-aged adults (aged 25–65 years), classified as low-to-moderate QRISK3 scores, underwent brain 3T diffusion magnetic resonance imaging (MRI) to evaluate ePVS burden and calculate DTI-ALPS indices. Processing speed index (PSI) was assessed using the Wechsler Adult Intelligence Scale—Version IV (WAIS-IV). **Results**: Approximately 43% of subjects reported having ePVS with significantly lower DTI-ALPS indices and PSI compared to those without ePVS. The DTI-ALPS index was inversely correlated with ePVS burden and positively correlated with PSI. Mediation analysis showed that the lower DTI-ALPS partially mediated the association between ePVS burden and slower processing speed. **Conclusions**: Visible ePVS in our cohort may reflect early glymphopathy and subtle cognitive slowing, while the DTI-ALPS index may serve as an early biomarker for preclinical CSVD-related cognitive vulnerability, supporting targeted prevention strategies.

## 1. Introduction

Cerebral small vessel disease (CSVD) represents a leading cause of vascular cognitive impairment and stroke, accounting for up to 45% of dementia cases globally [1]. Despite its profound societal burden, CSVD often progresses silently, particularly in its early stages, with white matter hyperintensities (WMHs) and enlarged perivascular spaces (ePVS) frequently detected incidentally on neuroimaging in asymptomatic adults [2,3]. These covert lesions are associated with subtle but insidious declines in processing speed, executive function, and gait stability, and their presence predicts increased risks of stroke, depression, and long-term functional dependency [4,5]. The subclinical nature of CSVD, coupled with its diffuse pathology and age-related progression, presents a formidable challenge to early diagnosis and timely intervention.

The current understanding of CSVD pathophysiology implicates a spectrum of microvascular dysfunctions, including endothelial injury, blood–brain barrier (BBB) disruption, chronic hypoperfusion, and low-grade neuroinflammation, which unfold over years before becoming radiologically or clinically apparent [6,7,8]. These processes eventually manifest as ePVS, WMHs, lacunes, and cerebral microbleeds, but they often begin in youth or middle age. Accordingly, there is growing interest in identifying early-stage surrogate markers that are non-invasive, quantifiable, and sensitive to disease-related changes before the onset of overt cognitive impairment.

ePVS has emerged as a promising radiological marker of early CSVD. These fluid-filled spaces surrounding penetrating arterioles facilitate the drainage of interstitial solutes and are increasingly understood as structural correlates of impaired waste clearance within the brain [9]. Their accumulation in strategic regions, such as the basal ganglia and centrum semiovale, is linked to neurovascular damage and poor cognitive outcomes [10]. However, the pathophysiological mechanisms underlying ePVS burden remain incompletely understood.

Recent studies have suggested that dysfunction of the glymphatic system or glymphopathy, a perivascular clearance network that facilitates the movement of cerebrospinal fluid (CSF) along para-arterial routes, may underlie the development of ePVS and other CSVD features [11]. A novel diffusion-based imaging marker, the diffusion tensor image analysis along the perivascular space (DTI-ALPS) index, has been proposed as a non-invasive surrogate or indirect measure of glymphatic function [12]. This index captures the ratio of water diffusivity along the perivascular space compared to surrounding axonal tracts. While DTI-ALPS is increasingly used in neurovascular and neurodegenerative research, its physiological specificity remains debated and may reflect not only fluid dynamics but also microstructural white matter integrity [12,13].

Moreover, cognitive changes in CSVD are heterogeneous, but slowing processing speed is among the earliest and most robust impairments observed, even in individuals who appear neurologically intact [14,15]. Processing speed deficits are thought to reflect the vulnerability of long-range white matter tracts to diffuse ischemic damage and axonal dysfunction, making them a useful proxy for early cognitive decline in CSVD. In parallel with neuroimaging and cognitive changes, cardio-cerebrovascular risk scores such as QRISK3 are increasingly employed to predict long-term cerebrovascular outcomes. The QRISK3 algorithm integrates conventional atherosclerotic risk factors, such as age, blood pressure, diabetes, and smoking, alongside medication use and psychosocial factors to estimate 10-year cardiovascular and cerebrovascular event risk [16,17]. While high QRISK3 scores have been associated with cerebral microangiopathy, even in asymptomatic adults [18], the neuroimaging correlates of low-to-moderate QRISK3 risk profiles remain underexplored. This raises the question of whether structural or functional brain changes associated with CSVD may already be detectable in these lower-risk populations.

Moreover, unlike most prior studies, which focused on older adults or clinical populations with established cerebrovascular disease, this study uniquely investigated these associations in a younger, working-age cohort with low-to-moderate scores on QRISK3. By doing so, this study aimed to determine whether subtle alterations in glymphatic-related diffusivity and processing speed can already be detected in ostensibly low-risk individuals, thereby extending the application of DTI-ALPS into a novel population. Specifically, this study examined whether lower DTI-ALPS index values, cautiously interpreted as a proxy for glymphopathy, may mediate the relationship between ePVS burden and slower processing speed. This work adds to ongoing efforts to characterize preclinical CSVD and to identify early neuroimaging markers that could inform risk stratification and prevention strategies.

## 2. Materials and Methods

### 2.1. Ethical Approval, Sample Size Estimation, and Subject Recruitment

This study was conducted at Hospital Universiti Sains Malaysia (HUSM) and received ethical approval from the Human Research Ethics Committee USM (USM/JEPeM/15030096). Subjects were recruited through convenience sampling among routine attendees of the Family Medicine Clinic. Demographic and clinical data were collected to calculate individual cardio-cerebrovascular risk scores using the QRISK3 algorithm.

**Figure 1:**
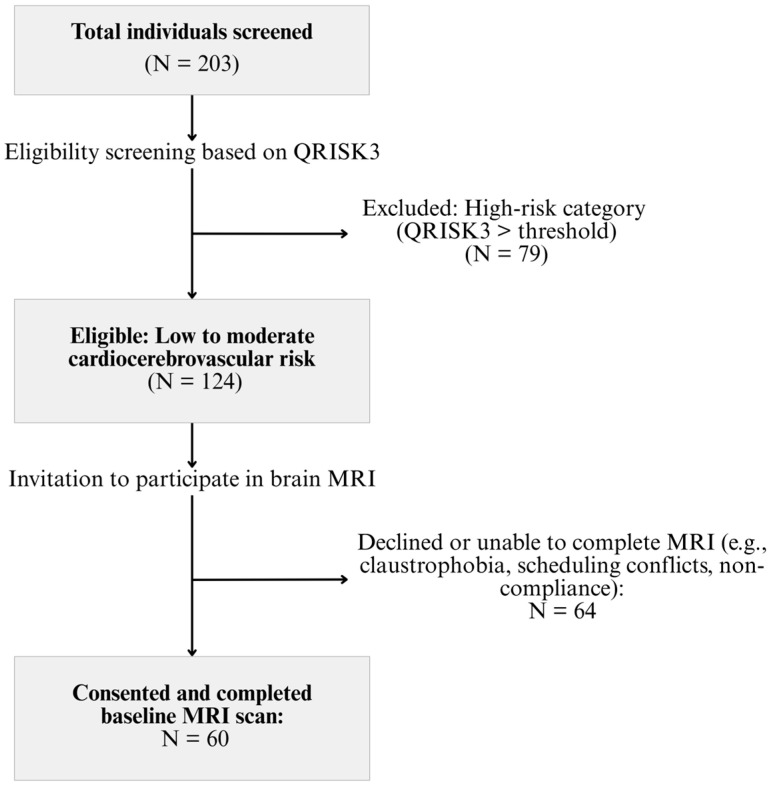
Study flowchart of subjects’ identification and inclusion/exclusion.

The target sample size was determined using G*Power (v3.1.9.7) (Heinrich-Heine-Universität, Düsseldorf, Germany), which indicated that 54 subjects would be needed to detect a moderate correlation (*r* = 0.35) between variables with an alpha level of 0.05 and 80% statistical power [19,20]. To accommodate potential attrition, 60 participants were enrolled. Of the 203 individuals screened, 124 met eligibility criteria, and 60 completed the MRI brain scan (Figure 1). This sample size was sufficient for correlational and multivariable analyses involving up to five predictors, adhering to the recommended minimum subject-to-variable ratio of 10:1 [12,20].

### 2.2. Cardio-Cerebrovascular Risk Assessment

Cardio-cerebrovascular risk was assessed using the QRISK3 (2018) online calculator (http://www.qrisk.org/index.php, accessed on 28 January 2024), developed by the University of Nottingham and EMIS. This tool predicts the 10-year risk of cardiovascular disease based on demographic and clinical factors. Risk categories were defined as low (≤10%), moderate (10.1–20%), and high (≥20.1%) according to established thresholds [21].

Eligible participants were asymptomatic young-to-middle-aged adults (aged 25–65 years) with no history of neurological disease and classified as having low-to-moderate cardio-cerebrovascular risk according to their QRISK3 scores. Individuals with older age, high cardiovascular risk (≥20.1% per QRISK3), or any prior neurological diagnosis were excluded to minimize confounding from established or subclinical cerebrovascular pathology that could independently influence white matter structure and cognitive performance. This approach aimed to improve cohort homogeneity and enhance sensitivity in detecting subtle, early microstructural brain changes associated with low-to-moderate cardio-cerebrovascular risk in an otherwise asymptomatic population. By excluding high-risk individuals, we sought to reduce bias from pre-existing vascular burden, silent infarcts, or prodromal neurodegenerative processes, thereby facilitating the identification of potential neuroimaging biomarkers relevant for early CSVD prevention and risk stratification [22].

### 2.3. Neurocognitive Assessment

Cognitive performance was assessed using the Wechsler Adult Intelligence Scale—Version IV (WAIS-IV) [23]. Three composite indices were analyzed to capture distinct cognitive domains relevant to white matter integrity: (i) the perceptual reasoning index (PRI), which assesses non-verbal reasoning and visuospatial problem solving, (ii) working memory index (WMI), reflecting attention, mental manipulation, and sequential processing, and (iii) processing speed index (PSI), which evaluates the speed of visual information processing and graphomotor coordination. Subtest scores from each index were used for multimodal analyses to explore associations with white matter integrity and cardio-cerebrovascular risk.

### 2.4. Diffusion MRI Protocols

A Philips Achieva (Best, The Netherlands) 3T MRI machine with a 32-channel head coil (*b*-value: 1000 s/mm^2^) was used for brain scanning with the following acquisition parameters: for structural image acquisition 3D-T1, echo time (TE)/repetition time (TR) = 10/678 ms, reconstruction matrix = 512 × 512 × 40, field of view (FOV) = 230 mm, voxel size = 0.45 mm × 0.45 mm, slice spacing = 0 mm, slice thickness = 2.5 mm, flip angle = 70, and 180 contiguous sagittal slices orientation; for T2, TE/TR = 80/3000 ms, reconstruction matrix = 512 × 512 × 24, FOV = 230 mm, voxel size = 0.45 × 0.45, slice spacing = 1.0 mm, slice thickness = 2.5 mm, and flip angle = 90; for 3D-fluid attenuated inversion recovery (FLAIR), TE/TR = 125/11,000 ms, inversion time (TI) = 2800 ms, reconstruction matrix = 512 × 512 × 24, FOV = 230 mm, voxel size = 0.45 mm × 0.45 mm, slice spacing = 0 mm, slice thickness = 2.5 mm, flip angle = 90, and 170 contiguous sagittal slices orientation; and for DTI, a scheme of 32 directions with a *b*-value of 1000 s/mm^2^, TE/TR = 72/6951 ms, reconstruction matrix = 96 × 96, FOV = 240 mm, voxel size = 2.5 mm × 2.5 mm, slice spacing = 0 mm, slice thickness = 2.5 mm, and flip angle = 90. The total scanner time was 15–20 min, per the subject’s ability.

### 2.5. ePVS Identification and Scoring

**Figure 2:**
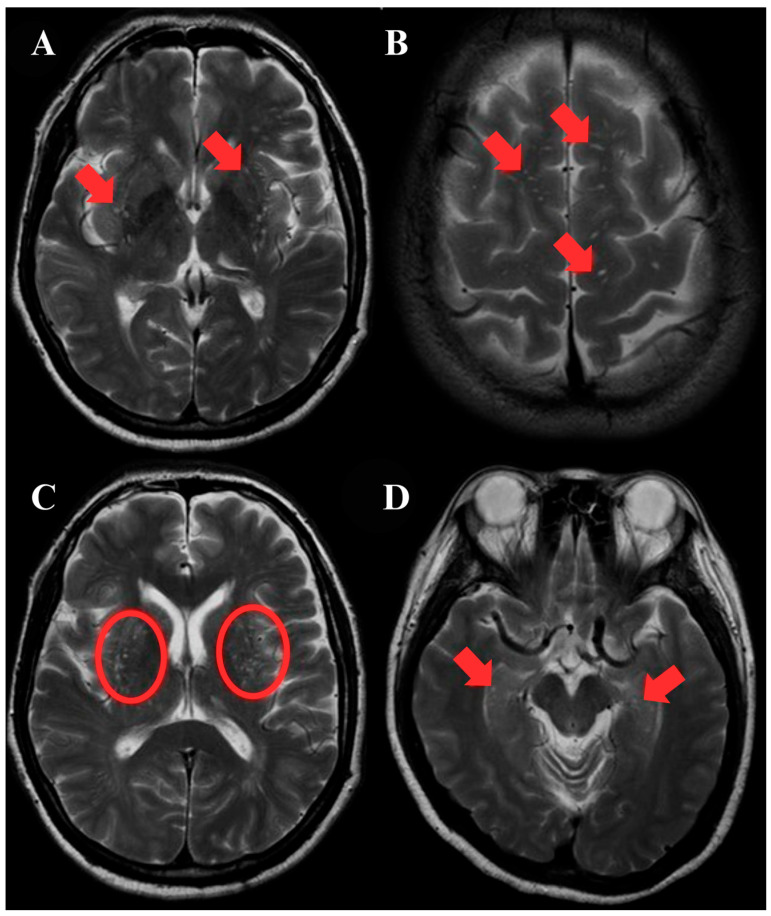
Structural T2-weighted images indicating the spatial distribution of enlarged perivascular spaces (ePVS) in subjects with ePVS. Images highlighting location (arrows indicate ePVS presence in different brain regions). Panels (**A**,**C**) refer to subjects showing more than one ePVS located exclusively in the basal ganglia at the globus pallidus, caudate nucleus, and putamen (red arrows in panel (**A**) and red circles in panel (**C**)). Panel (**B**) shows those subjects with multiple ePVS distributed in bi-hemispheric subcortical white matter, such as within the centrum semiovale. Panel (**D**) shows subjects with multiple ePVS at the bilateral hippocampus.

For each participant, the axial T2-weighted and FLAIR images exhibiting the greatest number of ePVS were independently selected in both the centrum semiovale and the basal ganglia, and their morphology was confirmed with T1 sequences, based on Standards for Reporting Vascular Changes on Neuroimaging 2 (STRIVE2) visual characteristics [8]. ePVS were identified according to the following criteria: (i) appearing as small, round or linear lesions depending on the imaging plane, with smooth and well-defined margins and typically measuring less than 3 mm in diameter; (ii) aligned with the expected course of perforating vessels; and (iii) demonstrating signal intensity equivalent to CSF across all MRI sequences (Figure 2). Counts were performed on three contiguous slices in each region with the greatest burden. Each region was graded using a semi-quantitative scale: 0 (no visible ePVS), 1 (1–10 ePVS), 2 (11–20 ePVS), 3 (21–40 ePVS), 4 (≥41 ePVS) [24,25]. Two neuroradiologists independently rated the images, blinded to the clinical data. The readings were performed in a randomized order, with at least one month in between to determine intra- and inter-rater agreement. Inter-rater agreement, as determined by kappa coefficients, was κ = 0.88 (95% CI: 0.8 to 1.0, *p* < 0.05), and intra-rater agreement was κ = 0.91 (95% CI: 0.8 to 1.0, *p* < 0.05).

### 2.6. DTI-ALPS Index Analysis

Raw DTI data were pre-processed using FMRIB Software Library (FSL version 5.0.9, https://fsl.fmrib.ox.ac.uk/fsl/docs/#/install/index, accessed on 6 October 2024). Pre-processing included brain extraction, correction for eddy current distortions and head motion, and voxel-wise calculation of diffusion metrics using *dtifit*, yielding fractional anisotropy (FA) and diffusivity values along the *x*-, *y*-, and *z*-axes (D*x*, D*y*, D*z*).

**Figure 3:**
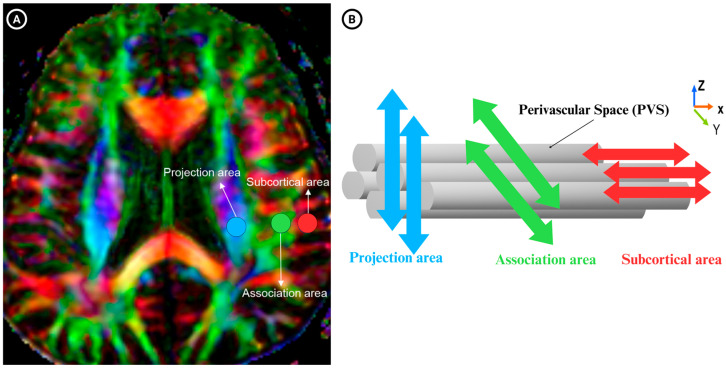
(**A**) illustrates three regions of interest (ROIs) positioned in areas containing projection fibers (projection area), association fibers (association area), and subcortical fibers (subcortical area) to assess diffusivities along the x, y, and z directions. (**B**) depicts the relationship between the orientation of the perivascular space (gray cylinders) and the orientation of the surrounding fibers.

The DTI-ALPS index calculation was based on the previous literature [26,27,28,29]. The axial slice at the level of the lateral ventricle body was selected (Figure 3A). At this level, the perivascular spaces are oriented perpendicular to the ventricular wall, corresponding predominantly to the right–left (*x*-axis) direction in the axial plane. This orientation is orthogonal to both the projection fibers (mainly along the *z*-axis) and the association fibers (mainly along the *y*-axis). Consequently, diffusivity along the x-axis in these regions partly reflects water movement along perivascular spaces. Spherical regions of interest (ROIs) with a 5 mm diameter (12 voxels each) were manually placed in the left hemisphere within the projection fibers, association fibers, and subcortical fibers (Figure 3B).

For each ROI, diffusivity values along the *x*-, *y*-, and *z*-axes were extracted, i.e., (i) D*x*-proj and D*y*-proj from the projection fiber ROI, (ii) D*x*-assoc and D*z*-assoc from the association fiber ROI. Since the perivascular spaces align along the *x*-axis, D*x*-proj and D*x*-assoc represent diffusivity parallel to the PVS, relatively independent of the dominant fiber orientation. The DTI-ALPS index was calculated as the ratio of mean diffusivity along the PVS to mean diffusivity perpendicular to the main fibers, using the following formula:

$$\mathrm{DTI}\text{-}\mathrm{ALPS}\text{-}\mathrm{index}\;=\;\frac{mean\;(Dx-proj,Dx-assoc)}{mean\;(Dy-proj,Dz-assoc)}$$

Diffusivity values were computed with 3D Slicer (version 4.1.1), which automatically aggregated voxel data within each ROI. All ROIs were delineated by two experienced radiologists blinded to participant information to minimize bias. To standardize ROI placement and minimize operator dependency, all raters underwent structured training based on a standard operating procedure (SOP) that specified anatomical landmarks, slice selection, and ROI size/placement. Training included practice cases and consensus sessions to harmonize interpretation. Quality control was performed by periodic cross-checking of ROI placement between raters, and any discrepancies were resolved through joint review. All ROIs were manually delineated in 3D Slicer, and rater performance was periodically audited against the SOP throughout the study.

### 2.7. Statistical Analysis

All statistical analyses were performed using IBM SPSS Statistics (version 26; IBM Corp, Armonk, NY, USA). Normally distributed variables are presented as mean ± standard deviation (SD) and non-normally distributed variables as median and interquartile range. Between-group comparisons were conducted using independent sample *t*-tests or Mann–Whitney U-tests, as appropriate. Categorical variables were compared using chi-squared tests or Fisher’s exact tests. Bivariate correlations between ePVS, DTI-ALPS index values, and cognitive scores were assessed using Pearson correlation coefficients. Logistic regression models were used to explore associations between ePVS presence (dichotomized as present/absent) and demographic or clinical predictors. Variance inflation factors (VIFs) were calculated to assess multicollinearity among covariates, with a threshold of VIF < 2 considered acceptable.

The analysis for the contour plot was performed using MINITAB 17 software (Minitab, LLC, Pennsylvania State University, State College, PA, USA) to determine the graphical relationship between one or more response variables and a set of quantitative experimental variables or factors [30]. To examine whether DTI-ALPS index values may mediate the relationship between ePVS burden and processing speed, mediation analyses were conducted using the PROCESS macro for SPSS (Model 4). Given the modest sample size (N = 60) relative to the complexity of mediation models with covariate adjustment, we acknowledge that the statistical power to detect small indirect effects may be limited. To partially address this, mediation effects were evaluated using bias-corrected bootstrap confidence intervals (10,000 resamples), which provide more robust estimates of indirect effects in smaller samples. All regression and mediation models were adjusted for age, sex, and QRISK3 score to account for potential confounding. A two-tailed *p*-value < 0.05 was considered statistically significant.

## 3. Results

### 3.1. Subjects’ Demographics and Characteristics

The demographics and characteristics of the study subjects are presented in Table 1. The mean age of the cohort was 38.47 ± 8.63 years. Of the 60 participants, 19 (31.7%) were male, and 41 (68.3%) were female. Most were of Malay ethnicity (90%) and were non-smokers (52; 86.7%).

### 3.2. ePVS

The spatial distribution of ePVS is depicted in Figure 2, indicating the most common locations. Approximately 26 (43.3%) subjects reported having silent CSVD manifested as ePVS. Based on Table 1, subjects with ePVS were significantly older (mean age 43.1 ± 12.2, *p* = 0.02) and had higher systolic blood pressure (SBP) (*p* = 0.01), higher body mass index (BMI) (26.4 ± 3.6 kg/m^2^, *p* < 0.01), and higher QRISK3 scores (4.5% ± 5.9 vs. 1.3% ±1.7, *p* = 0.01) compared to those without ePVS. A family history of cardiovascular disease was more common in the ePVS group (46.2% vs. 8.8%, *p* = 0.004), as was hypertension (30.7% vs. 2.9%, *p* = 0.01). There were no significant differences between groups in sex distribution, ethnicity, smoking status, cholesterol to HDL ratio, or WAIS-IV cognitive indices. Although participants with ePVS had numerically lower PSI scores, this difference did not reach statistical significance (mean difference = −6.4 points, *p* = 0.08). The standardized mean difference was Cohen’s *d* = −0.41 (95% CI: −0.88 to 0.06), indicating a small-to-moderate, but statistically non-significant, effect size.

### 3.3. Risk Factors Associated with the Presence of ePVS

Table 2 presents the results of the multiple logistic regression analysis examining factors associated with the presence of ePVS. After adjustment for age and sex, several variables were significantly associated with higher odds of ePVS. A family history of cardiovascular disease (OR = 9.53, 95% CI: 1.86–48.91, *p* = 0.01) and hypertension (OR = 12.0, 95% CI: 1.38–104.34, *p* = 0.02) markedly increased the likelihood of ePVS. Conversely, higher BMI (OR = 0.83 per unit, *p* = 0.01), higher SBP (OR = 0.95 per mmHg, *p* = 0.02), and higher QRISK3 scores (OR = 0.75 per percentage point, *p* = 0.03) were associated with lower odds of ePVS, although the direction of these associations may reflect collinearity or sampling variability. Among cognitive measures, only the PSI was significantly related to ePVS, with higher PSI scores modestly increasing the odds (OR = 1.06, 95% CI: 1.00–1.13, *p* = 0.04). No significant associations were observed for the PRI and WMI.

All VIF values were <2, indicating that multicollinearity did not meaningfully affect model stability. While some predictors (e.g., BMI, SBP, QRISK3) exhibited sign reversals between univariate and multivariate models, these patterns likely reflect suppression effects and sampling variability in a modest cohort. Effect sizes expressed as odds ratios per SD confirmed the robustness of the main associations. Sensitivity analyses excluding outliers or adjusting model specifications yielded consistent results.

### 3.4. Relationship Between ePVS with the Risk Factors and Neurocognitive Profiles

**Figure 4:**
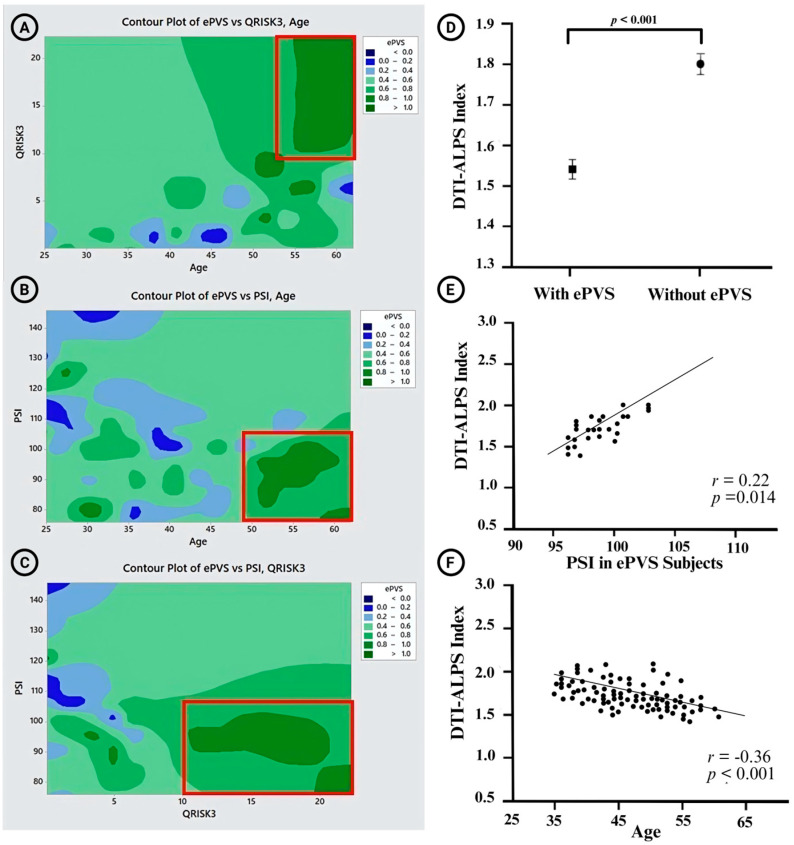
(**A**–**C**) Contour plots illustrating the distribution of enlarged perivascular spaces (ePVS) burden in relation to QRISK3 score, processing speed index (PSI), and age. (**A**) Higher ePVS burden was observed in individuals with elevated QRISK3 scores and older age (red box). (**B**) Increased ePVS burden clustered in participants with lower PSI and older age (red box). (**C**) Greater ePVS burden was seen in individuals with higher QRISK3 scores and lower PSI (red box). (**D**) Comparison of diffusion tensor image analysis along the perivascular space (DTI-ALPS) indices between subjects with and without ePVS, showing significantly higher indices in those without ePVS (*p* < 0.001). (**E**) Positive correlation between PSI and DTI-ALPS index in subjects with ePVS (*r* = 0.22, *p* = 0.014). (**F**) Negative inverse correlation between age and DTI-ALPS index (*r* = −0.36, *p* < 0.001).

Table 2 shows the bivariate correlations (*r*) and multivariable linear regression models with standardized regression coefficients (β) quantifying the associations between vascular risk factors and the presence of ePVS. Among all participants, several risk factors demonstrated moderate associations with ePVS status. Age (*r* = 0.30), SBP (*r* = 0.32), and QRISK3 score (*r* = 0.35) were positively correlated with the presence of ePVS. The contour plot shows the distribution of ePVS burden in relation to QRISK3 score and age (Figure 4A). Higher ePVS are predominantly observed in individuals with higher QRISK3 scores (>15) and older age (>55 years). In multivariable linear regression models adjusting for age, sex, and QRISK3 score, higher SBP (β = 0.27) and higher QRISK3 score (β = −0.30) remained significant predictors.

WAIS-IV PSI showed an inverse correlation (*r* = −0.42), indicating that lower processing speed was associated with ePVS. The adjusted association for WAIS-IV PSI also remained substantial (β = 0.38). The contour plot (Figure 4B) illustrates ePVS burden in relation to PSI and age. Increased ePVS burden clustered among participants with lower PSI scores (<105) and older age (>55 years). In participants without ePVS, none of the risk factors demonstrated notable correlations, with absolute *r*-values ranging from −0.12 to 0.20 and no significant regression coefficients. Moreover, the contour plot (Figure 4C) depicts the joint distribution of ePVS burden with QRISK3 score and PSI. Higher ePVS levels were observed in individuals with both higher QRISK3 scores (>15) and lower PSI scores (<105).

### 3.5. DTI-ALPS Index

A general linear model was used to assess the DTI-ALPS index differences between subjects with and without ePVS, adjusting for sex, age, and hypertension as covariates. Subjects without ePVS reported having a significantly higher DTI-ALPS index (1.80 ± 0.15) compared to those with ePVS (1.55 ± 0.30). Post hoc analysis confirmed a significant difference between the two groups (*p* < 0.001) after Bonferroni corrections (Figure 4D).

In subjects with ePVS, the DTI-ALPS index showed a significant positive correlation with the PSI (r = 0.22, *p* = 0.014) (Figure 4E). However, no significant relationships were identified between the DTI-ALPS index and other cognitive domains. Moreover, there was a negative correlation between age and the DTI-ALPS index in subjects with ePVS (*r* = −0.463, *p* = 0.005). For the overall sample, the correlation coefficient between age and the DTI-ALPS index was −0.346 (*p* < 0.001) (Figure 4F). However, no significant correlation was observed between age and the DTI-ALPS index in subjects without ePVS (*p* = 0.304). Additionally, no notable associations were identified between the DTI-ALPS index and other demographic factors.

### 3.6. Mediating Role of the DTI-ALPS Index

**Figure 5:**
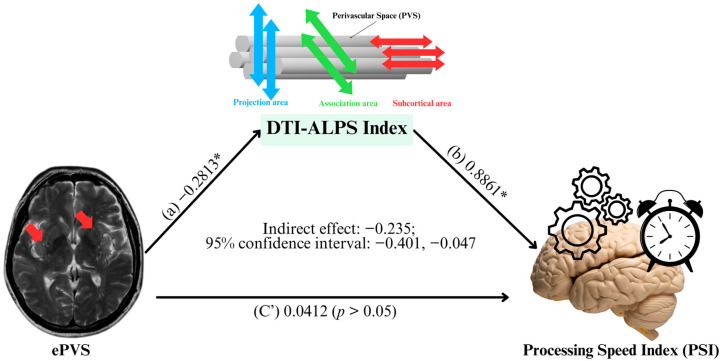
The mediation model illustrating the indirect association between enlarged perivascular space (ePVS) (red arrow) and processing speed index (PSI) via diffusion tensor imaging along the perivascular space (DTI-ALPS) index. The presence of ePVS was significantly associated with lower DTI-ALPS index (**path a**). In turn, lower DTI-ALPS index was associated with reduced PSI (**path b**). The indirect effect of ePVS on PSI via DTI-ALPS index was significant (indirect effect = −0.235; 95% CI: −0.401 to −0.047), while the direct effect (**path c′**) was non-significant (β = 0.0412, *p* > 0.05), indicating partial mediation. The asterisks denote * *p* = significant difference (two-tailed) at the 0.05 level.

Figure 5 illustrates the mediation analysis testing whether the DTI-ALPS index mediates the association between ePVS and PSI. The presence of ePVS was significantly associated with lower DTI-ALPS index (path a: β = −0.2813, *p* < 0.05). In turn, higher DTI-ALPS index was positively associated with PSI (path b: β = +0.8861, *p* < 0.05). The indirect effect of ePVS on PSI through DTI-ALPS was statistically significant (indirect effect = −0.235, 95% CI: −0.401 to −0.047, T-statistic: −2.61), indicating partial mediation. The direct association between ePVS and PSI after accounting for the DTI-ALPS index (path c′) was non-significant (β = 0.0412, *p* > 0.05), suggesting that the observed relationship between ePVS and processing speed was largely explained by variation in DTI-ALPS index.

## 4. Discussion

This study provides new insights into the potential role of glymphatic dysfunction (or glymphopathy) in cognitive performance among asymptomatic, working-age individuals with imaging evidence of CSVD. ePVS, also known as Virchow–Robin spaces, are increasingly recognized as a sensitive neuroimaging marker of CSVD. These fluid-filled spaces surrounding penetrating vessels are frequently observed in conjunction with other small vessel disease features, including WMHs, lacunar infarcts, and cerebral atrophy [8]. Notably, ePVS often localize adjacent to WMH clusters in frontal, parietal, and temporal regions and are associated with silent lacunar strokes and cortical atrophy, reflecting a shared underlying microvascular pathology [31]. Beyond structural correlates, ePVS burden has been linked to cognitive impairment, particularly slowed processing speed, even after accounting for WMHs and lacunes [32,33]. The proposed mechanisms include glymphopathy, leading to accumulation of neurotoxic metabolites, neuroinflammation, and BBB dysfunction [34,35]. Moreover, the established risk factors for ePVS were also widely described, including advancing age, hypertension, and genetic susceptibility, such as the apolipoprotein E (APOE)-ε4 allele [36,37,38].

Against this backdrop, our findings extend prior work by demonstrating that in a relatively young, low-to-moderate vascular risk cohort, as determined by QRISK3 scores, there were early alterations in perivascular diffusivity in over 40% of participants. Specifically, higher ePVS burden was associated with lower DTI-ALPS index and reduced processing speed. This association supports the hypothesis that perivascular enlargement may reflect early glymphopathy, with downstream effects on cognitive processing [39,40]. Our results also suggest that DTI-ALPS alterations can be detected in ostensibly healthy adults well before clinical symptoms become apparent, highlighting the potential of advanced diffusion imaging as a sensitive marker of preclinical CSVD-related changes [41,42]. Such early detection capability may hold promise for identifying preclinical CSVD-related changes and refining risk-stratification strategies. Our findings further reinforce the association between higher ePVS burden and reduced processing speed, a cognitive domain recognized as particularly vulnerable to white matter disruption [10,43]. Importantly, however, the DTI-ALPS index remains an indirect surrogate marker, and its physiological specificity and sensitivity are still debated [13]. Therefore, caution is warranted when interpreting this metric as a direct indicator of glymphopathy. The mediation analysis should be interpreted as exploratory and hypothesis-generating. Given the modest sample size and cross-sectional design, these findings do not establish causal pathways but rather provide preliminary evidence to support further longitudinal research.

Additionally, we did not observe significant associations between the DTI-ALPS index and executive function or working memory. This pattern likely reflects the differential susceptibility of cognitive domains to distinct CSVD pathologies [44,45]. Executive dysfunction, for example, may depend more on the burden and spatial distribution of WMHs, lacunes, or microinfarcts rather than solely on perivascular clearance disturbances [44,46]. Prior studies have demonstrated that executive processes rely on the integrity of fronto-subcortical circuits and extensive associative white matter tracts, such as the superior longitudinal fasciculus and anterior thalamic radiation, which are particularly vulnerable to diffuse ischemic injury [47,48,49]. In contrast, processing speed is thought to be more sensitive to early disruptions in microstructural connectivity and glymphatic flow, as reflected by DTI-ALPS alterations [50]. Moreover, the heterogeneity of executive function measures and the relatively preserved working memory scores observed in our cohort may have limited our ability to detect subtle associations. These observations underscore the need for larger studies employing more comprehensive neuropsychological batteries and advanced imaging modalities to disentangle the relative contributions of different CSVD markers to domain-specific cognitive impairment.

It is particularly notable that, despite the absence of overt neurological symptoms, some individuals with ePVS in our cohort exhibited DTI-ALPS index values comparable to those reported in individuals with symptomatic CSVD [51,52]. This observation reinforces the concept of a “silent phase” of microvascular pathology during which structural and functional brain changes may accumulate long before clinical impairment becomes detectable. Such subclinical alterations in glymphatic function may represent an early manifestation of small vessel disease and, if left unrecognized, could progress to more extensive tissue damage and cognitive decline. Moreover, given the increasing recognition of CSVD as a leading contributor to vascular cognitive impairment and dementia in older adults [1], the identification of early biomarkers, such as the DTI-ALPS index, may offer critical opportunities for intervention during this vulnerable preclinical window. These findings underscore the importance of routine monitoring in at-risk individuals, even those who are relatively young or clinically asymptomatic, as part of broader strategies aimed at preventing long-term cognitive deterioration associated with CSVD.

Nevertheless, several limitations must be considered. First, the cross-sectional design precludes any causal inference regarding the temporal relationship between ePVS burden, glymphopathy, and cognitive decline. Longitudinal studies will be essential to establish whether reduced DTI-ALPS index precedes cognitive impairment or merely reflects accumulating small vessel damage. Second, although ePVS was rated according to STRIVE2 criteria, observer bias remains possible. Future studies employing automated segmentation methods could improve reproducibility. Third, while the DTI-ALPS index is increasingly used as a non-invasive proxy for glymphatic function, it is also influenced by other factors, including white matter microstructure and CSF dynamics [13]. Complementary imaging modalities, such as dynamic contrast-enhanced MRI, would strengthen mechanistic inferences.

Furthermore, the present study employed a convenience sample drawn from a single health system and restricted eligibility to individuals with low-to-moderate QRISK3 scores. While this approach reduced confounding from advanced vascular pathology and improved cohort homogeneity, it inevitably introduced selection bias and limited external validity. Our participants may not be representative of the general population or of higher-risk groups in whom cerebrovascular pathology and glymphatic dysfunction may be more pronounced. We did not have a sufficient number of high-QRISK3 individuals to conduct a meaningful sensitivity analysis including this subgroup. Future multicenter studies with broader recruitment criteria and stratified sampling across the full spectrum of vascular risk are needed to confirm whether the present findings generalize to more diverse and higher-risk populations.

Moreover, our sample size was modest (n = 60) although comparable to prior neuroimaging studies employing DTI-ALPS [12,53,54]. Larger, more diverse cohorts will be required to improve generalizability and clarify domain-specific cognitive associations. Moreover, our cognitive assessment was limited to WAIS-IV indices, including the PSI, PRI, and WMI, without incorporating a broader battery of executive function tests, such as set-shifting, inhibition, or verbal fluency. Given that executive dysfunction is a hallmark of CSVD, the inclusion of these measures would have provided a more comprehensive evaluation of domain-specific cognitive vulnerability. Although PSI subtest-level scores (Coding and Symbol Search) were available, we did not analyze them separately because our study was designed to investigate associations at the index level, which provides a validated and reliable composite measure of processing speed. This approach was considered sufficient to address our objectives while avoiding the inflation of Type I error due to multiple comparisons. Nonetheless, future studies could examine subtest-level performance to explore whether distinct aspects of processing speed are differentially affected in early CSVD.

Finally, differences between our cohort and the broader local or national population, such as age distribution, sex ratio, or prevalence of vascular risk factors, could have influenced the observed associations. Some coefficient sign reversals observed between univariate and multivariate models likely reflect suppression effects or sample variability rather than biologically protective associations. While VIF values suggested that collinearity was minimal, these patterns underscore the need for replication in larger datasets where feasible. Future work should include comparative demographic tables or benchmark recruitment data against population-level statistics to better assess external validity.

The strengths of this study include its focus on an underrepresented population—clinically asymptomatic, working-age adults—and the integration of advanced diffusion imaging with established cognitive measures. Whereas most prior investigations of ePVS, glymphatic function, and cognition have centered on older or symptomatic populations, our results demonstrate that such associations may already be present in community-dwelling adults well before overt clinical manifestations. By demonstrating that DTI-ALPS index values are associated with processing speed in this cohort, our findings suggest that glymphopathy may be detectable well before clinical impairment emerges. However, this work should be interpreted as exploratory, and further research using longitudinal designs and broader neuropsychological batteries is warranted to confirm and extend these observations.

Taken together, our results point to a potential role of glymphopathy, as estimated by the DTI-ALPS index, as an early marker of subclinical cognitive vulnerability associated with ePVS burden in silent CSVD. While the index shows promise as a research tool, its utility for screening and intervention remains to be established. Future studies should integrate advanced neuroimaging and glymphatic assessment into longitudinal designs to clarify the mechanistic pathways linking microvascular disease to cognitive decline and to evaluate whether glymphatic-targeted therapies may mitigate CSVD progression.

## 5. Conclusions

This study provides preliminary evidence that a manifestation of silent CSVD (i.e., ePVS) and reduced DTI-ALPS index values—an indirect marker of glymphatic dysfunction (or glymphopathy)—are associated with slower processing speed in asymptomatic, community-dwelling adults. These findings may open new insight that microvascular and glymphatic alterations may occur well before overt neurological symptoms emerge. However, given that DTI-ALPS is not a direct measure of glymphatic function and that the cross-sectional design precludes causal inference, these relationships should be interpreted as hypothetical rather than definitive. Moreover, while the cross-sectional design limits causal inference, the results underscore the potential utility of advanced diffusion imaging as a sensitive biomarker of early CSVD-related brain changes. Given the cross-sectional design, future longitudinal studies incorporating comprehensive neuropsychological assessments and multimodal imaging will be essential to clarify the temporal dynamics of glymphopathy, to establish its prognostic value, and to evaluate whether targeting glymphatic pathways can mitigate cognitive decline in individuals at risk of CSVD-related dementia.

## Figures and Tables

**Table 1:** Demographics and characteristics of the study subjects.

Variables	Total (N = 60)	ePVS		
		Present (n = 26)	Absent (n = 34)	*p*-Value
Age, yrs *	38.47 ± 8.63	43.12 ± 12.2	36.3 ± 10.1	0.02
Sex, n (%)				0.22
Male	19 (31.7)	10 (38.5)	9 (26.4)	
Female	41 (68.3)	16 (61.5)	25 (73.6)	
Ethnicity, n (%)				0.63
Malay	54 (90)	24 (92.4)	30 (88.2)	
Chinese	4 (6.7)	1 (3.8)	3 (8.8)	
Others	2 (3.3)	1 (3.8)	1 (3)	
Education level, n (%)				0.54
Low ^a^	5 (8.3)	3 (11.5)	2 (5.9)	
Intermediate ^b^	27 (45)	12 (46.2)	15 (44.1)	
High ^c^	28 (46.7)	11 (42.3)	17 (50)	
Smoking, n (%)				0.35
Non-smoker	52 (86.6)	22 (84.6)	30 (88.2)	
Ex-smoker	6 (10)	3 (11.5)	3 (8.8)	
Light smoker	1 (1.7)	0 (0)	1 (3.0)	
Moderate smoker	1 (1.7)	1 (3.9)	0 (0)	
Heavy smoker	0 (0)	0 (0)	0 (0)	
Type 2 diabetes, n (%)	0 (0)	0 (0)	0 (0)	-
Family history ^d^, n (%)	15 (25)	12 (46.2)	3 (8.8)	0.004
Atrial fibrillation, n (%)	0 (0)	0 (0)	0 (0)	-
Hyperlipidemia, n (%)	0 (0)	0 (0)	0 (0)	-
Hypertension, n (%)	9 (25)	8 (30.7)	1 (2.9)	0.01
CHO to HDL ratio *	3.80 ± 0.39	3.97 ± 0.88	3.80 ± 1.09	0.52
SBP (mmHg) *	130.47 ± 10.5	134.3 ± 15.8	123.9 ± 13.5	0.01
BMI (kg/m^2^) *	25.06 ± 1.51	26.4 ± 3.6	23.2 ± 4.6	*p* < 0.001
QRISK3 score (%) *	2.85 ± 3.42	4.5 ± 5.9	1.3 ± 1.7	0.01
WAIS-IV indices *				
PRI	100.2 ± 4.29	101.7 ± 12.8	102.7 ± 12.3	0.80
WMI	110.1 ± 4.01	107.4 ± 18.5	108.5 ± 19.6	0.85
PSI	101.5 ± 5.20	98.7 ± 12.7	105.1 ± 14.5	0.08

Note: Data values are presented as number of subjects (n), with percentage (%) in parentheses. Light smoker (≤10); Moderate smoker (10–19); Heavy smoker (≥20); * Data are means ± standard deviations; ^a^ None, primary or secondary education; ^b^ Higher secondary or vocational; ^c^ University or higher professional education; ^d^ Angina or heart attack in a first-degree relative <60 years of age, n (%). Subgroups according to the presence or absence of enlarged perivascular spaces (ePVS) were compared using χ^2^ test and independent sample *t*-test (*p* = significant difference [two-tailed] at the < 0.05 level). BMI, body mass index; CHO, cholesterol; HDL, high-density lipoprotein; PRI, perceptual reasoning index; PSI, processing speed index; SBP, systolic blood pressure; WAIS-IV, Wechsler Adult Intelligence Scale—Version IV; WMI, working memory index.

**Table 2:** Relationship between enlarged perivascular spaces (ePVS) with the risk factors and neurocognitive profiles.

Risk Factors	Multiple Logistic Regression ^a^ (With vs. Without)			*r (*β)		VIF
	OR (95% CI)	OR per 1 SD (95% CI)	*p*-Value *	With ePVS	Without ePVS	
Age ^b^	0.95 (0.89 to 0.99)	1.32 (1.05–1.65)	0.03	0.30 * (−0.25)	0.10 (−0.05)	1.12
Family history ^c^	9.53 (1.86 to 48.91)	–	0.01	−0.25 (−0.20)	−0.05 (−0.02)	1.05
Hypertension	12.0 (1.38 to 104.34)	–	0.02	−0.28 (−0.22)	−0.08 (−0.04)	1.08
Systolic blood pressure ^d^	0.95 (0.91 to 0.99)	0.84 (0.72–0.98)	0.02	0.32 * (0.27 *)	0.12 (0.08)	1.25
Body mass index	0.83 (0.72 to 0.96)	0.76 (0.63–0.91)	0.01	−0.20 (−0.15)	0.05 (0.03)	1.18
QRISK3 score	0.75 (0.58 to 0.98)	0.81 (0.66–0.98)	0.03	0.35 * (−0.30 *)	−0.10 (−0.07)	1.31
WAIS-IV PRI	0.98 (0.92 to 1.05)	0.96 (0.87–1.05)	0.53	0.05 (0.03)	0.10 (0.05)	1.07
WAIS-IV WMI	0.99 (0.94 to 1.03)	0.97 (0.90–1.04)	0.52	0.10 (0.08)	0.12 (0.10)	1.06
WAIS-IV PSI	1.06 (1.00 to 1.13)	1.15 (1.01–1.32)	0.04	−0.42 * (0.38 *)	0.20 (0.15)	1.14

CI, confidence interval; ePVS, enlarged perivascular spaces; IQR, interquartile range; OR, odds ratio; PRI, perceptual reasoning index; PSI, processing speed index; WAIS-IV, Wechsler Adult Intelligence Scale—Version IV; WMI, working memory index. ^a^ Adjusted for age and gender; ^b^ Per one year; ^c^ Angina or heart attack in a first-degree relative < 60 years of age; ^d^ Per mmHg; r, Pearson correlation coefficient; β, multivariate unstandardized beta coefficient; * *p* = significant difference (two-tailed) at 0.05 level.

## Data Availability

The datasets generated and/or analyzed during the current study can be made available from the corresponding author upon reasonable request.

## Abbreviations

The following abbreviations are used in this manuscript:

APOE	Apolipoprotein E
BBB	Blood–brain barrier
CHO	Cholesterol
CSF	Cerebrospinal fluid
CSVD	Cerebral small vessel disease
DTI-ALPS	Diffusion tensor image analysis along the perivascular space
ePVS	Enlarged perivascular spaces
FA	Fractional anisotropy
FLAIR	Attenuated inversion recovery
FOV	Field of view
HDL	High-density lipoprotein
HUSM	Hospital Universiti Sains Malaysia
MRI	Magnetic resonance imaging
PRI	Perceptual reasoning index
PSI	Processing speed index
ROI	Region of interest
SBP	Systolic blood pressure
STRIVE-2	Standards for Reporting Vascular Changes on Neuroimaging 2
TE	Echo time
TI	Inversion time
TR	Repetition time
WAIS-IV	Wechsler Adult Intelligence Scale—Version IV
WMHs	White matter hyperintensities
WMI	Working memory index
